# Advances and Applications of Spatial Proteomics: From Organellar Maps to Clinical Translation

**DOI:** 10.1002/cbic.202500616

**Published:** 2026-01-14

**Authors:** Chiara Bernardini, Maike Däther, Franziska R. Traube

**Affiliations:** ^1^ Institute of Biochemistry Universität Stuttgart Allmandring 31 70569 Stuttgart Germany; ^2^ Faculty of Chemistry and Pharmacy Ludwig‐Maximilians‐Universität München Butenandtstr. 5–13 81377 Munich Germany; ^3^ TUM School of Natural Sciences Technical University of Munich Lichtenbergstraße 4 85748 Garching Germany

**Keywords:** fractionation, mass spectrometry, microscopy‐guided segmentation, organelle profiling, protein translocations, proteome profiling

## Abstract

Spatial proteomics has emerged as a powerful approach to systematically map the subcellular localization of thousands of proteins in parallel, providing insights into organelle composition, protein trafficking, and context‐dependent relocalization events. Building on advances in mass spectrometry sensitivity, and acquisition as well as quantification strategies, organelle‐resolved protein maps can now be generated with unprecedented depth and resolution, and recent workflows have expanded the applicability of spatial proteomics to diverse experimental and challenging contexts. Complementary bioinformatic pipelines enable the assignment of proteins to compartments, the detection of distribution shifts, and the integration of spatial data with other omics layers. Beyond fundamental cell biology, the technology holds great potential for clinical research, where limited input material and the complexity of primary samples pose specific challenges. Emerging low‐input preparation methods, antibody‐based organelle enrichment, and microscopy‐guided approaches offer promising solutions, while robust, marker‐independent data analysis will be essential to handle the biological variability of patient‐derived samples. As protocols become more automated, low‐input compatible, and bioinformatically standardized, spatial proteomics is poised to become a valuable tool for mechanistic disease research, biomarker discovery, and therapeutic target identification.

## Graphical abstract

Spatial proteomics provides high‐resolution maps of protein localization and trafficking by combining advanced mass spectrometry with complementary imaging and computational tools. Emerging low‐input and automated workflows are expanding its applicability to challenging clinical samples, supporting mechanistic disease research and biomarker discovery.

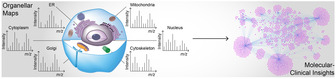

## 1 Cellular Compartmentalization and Its Clinical Relevance

Over the past decade, omics technologies have substantially advanced our understanding of cellular organization and molecular processes, enabling systematic characterization of genome mutations, gene expression patterns, protein concentrations, and metabolic states in cells in both physiological and pathological contexts.^[^ ^1^ ^,^ ^2^ ^]^ Next‐generation sequencing (NGS) has transformed clinical diagnostics, and mass spectrometry (MS)‐based proteomics is increasingly integrated into diagnostic workflows.^[^ ^3^, ^4^, ^5^ ^]^ Leveraging advances in artificial intelligence (AI), these approaches reveal complex molecular relationships underlying disease progression.^[^ ^6^ ^]^ However, global omics data alone often cannot fully capture the phenotypic state of a cell, as cellular function critically depends on context‐specific molecular interactions and their subcellular localization, which remain only partially resolved by conventional omics methods, including NGS.^[^ ^7^ ^]^

Eukaryotic cells exhibit a high degree of spatial organization. Subcellular compartments such as the nucleus, cytosol, mitochondria, endoplasmic reticulum (ER), Golgi apparatus, and lysosomes create defined microenvironments that support specific biochemical reactions and the permeability of the individual compartments is tightly regulated, in particular by different membrane compositions.^[^ ^8^ ^]^ For instance, the inner mitochondrial membrane enables proton‐gradient formation for ATP synthesis, whereas the nuclear envelope allows selective molecular transport of biomolecules as big as natively folded proteins through nuclear pore complexes.^[^ ^9^, ^10^, ^11^ ^]^ Some organelles, such as lysosomes, are highly dynamic and can form or degrade rapidly, whereas others, like the nucleus and mitochondria, are more stable but still undergo regulated remodeling during processes such as mitosis and mitochondrial biogenesis.^[^ ^12^, ^13^, ^14^ ^]^ Moreover, high molecular crowding within the cytosol restricts diffusion, emphasizing that not only the presence but also the precise localization of a protein determines its function.^[^ ^15^ ^]^ Thus, spatially resolved proteomic information is indispensable for understanding cellular function and dysfunction and aberrant protein localization is increasingly recognized as a key factor in disease initiation and progression.^[^ ^16^ ^]^ Mislocalization may result from mutations disrupting or generating transport signals, from defects in carrier proteins mediating translocation, or from alterations in posttranslational modifications (PTMs) that regulate compartment‐specific targeting.^[^ ^17^, ^18^, ^19^, ^20^, ^21^ ^]^

Many studies in clinical contexts have focused on proteins with altered nuclear distribution patterns, where two main categories can be distinguished. First, proteins that normally perform essential, constitutive functions in the nucleus but are excluded from the nucleus under pathological conditions and accumulate in the cytosol. Second, proteins that only translocate to the nucleus in specific contexts under physiological conditions, for example, transcription factors regulating pluripotency genes. Persistent nuclear localization of such factors can promote malignant transformation and enable unlimited proliferative potential.^[^ ^19^ ^,^ ^22^ ^,^ ^23^ ^]^ Mutations affecting nuclear localization signals (NLS) or nuclear export signals (NES) are frequent causes of such defects and can be readily detected in the clinical context by NGS.^[^ ^17^ ^,^ ^24^ ^,^ ^25^ ^]^ A classic example is Nucleophosmin 1 (NPM1), which shuttles between the nucleus and cytoplasm but is mainly nucleolar in untransformed cells.^[^ ^26^ ^,^ ^27^ ^]^ NPM1 mutations that cause loss of nucleolar localization and gain of an NES, resulting in cytoplasmic accumulation and loss of nuclear functions, are drivers of leukemogenesis in the context of acute myeloid leukemia (AML).^[^ ^17^ ^,^ ^24^ ^]^ Similar mechanisms underlie cytoplasmic mislocalization of the RNA‐binding protein FUS/TLS and the TAR DNA‐binding protein 43 (TDP‐43) in amyotrophic lateral sclerosis.^[^ ^25^ ^]^ In Alzheimer's disease, impaired nucleocytoplasmic transport due to defective nucleoporins was reported to facilitate cytoplasmic aggregation of the microtubule‐associated protein TAU.^[^ ^19^ ^]^

Defective PTM‐dependent targeting can also drive disease. For instance, a phosphorylation‐site mutation in the transcription factor GATAD1 disrupts 14‐3‐3 protein binding, leading to aberrant nucleocytoplasmic localization and contributing to dilated cardiomyopathy.^[^ ^21^ ^]^ In contrast, dysregulated protein turnover can also result in pathological nuclear accumulation, as observed for *β*‐catenin, a key effector of canonical WNT signaling.^[^ ^28^ ^]^ Normally, cytosolic *β*‐catenin is continuously degraded, but mutations in its degradation machinery cause nuclear stabilization and activation of oncogenic targets such as c‐MYC, promoting tumorigenesis.^[^ ^29^ ^]^

Furthermore, insights into subcellular protein distribution also offer important implications for infectious diseases and their impact on host cells, including potentially persistent alterations that extend beyond the acute phase of infection. For example, proteome‐wide profiling of human coronavirus OC43 infection revealed that only about 10% of proteins showing altered localization also changed in total abundance, demonstrating that abundance and localization are largely regulated independently.^[^ ^30^ ^]^ Such findings underscore the importance of dynamic localization mapping for understanding cellular responses to infection and stress, revealing information that cannot be captured by transcriptomic or total proteome analyses alone.

Altogether, these examples illustrate that subcellular organization is not a static architectural feature but a dynamic regulatory layer of cell biology. A holistic understanding of protein localization dynamics is therefore essential for elucidating disease mechanisms and developing targeted therapeutic strategies. This review is intended for a broad audience, including researchers who have never worked with proteomics, and aims to provide a comprehensive overview of spatial proteomics methods but also of exemplary applications where this technique offers advantages over global omics or targeted approaches.

## 2 Beyond One‐Way Trafficking: The Dynamic Nature of Protein Localization

**Figure 1:**
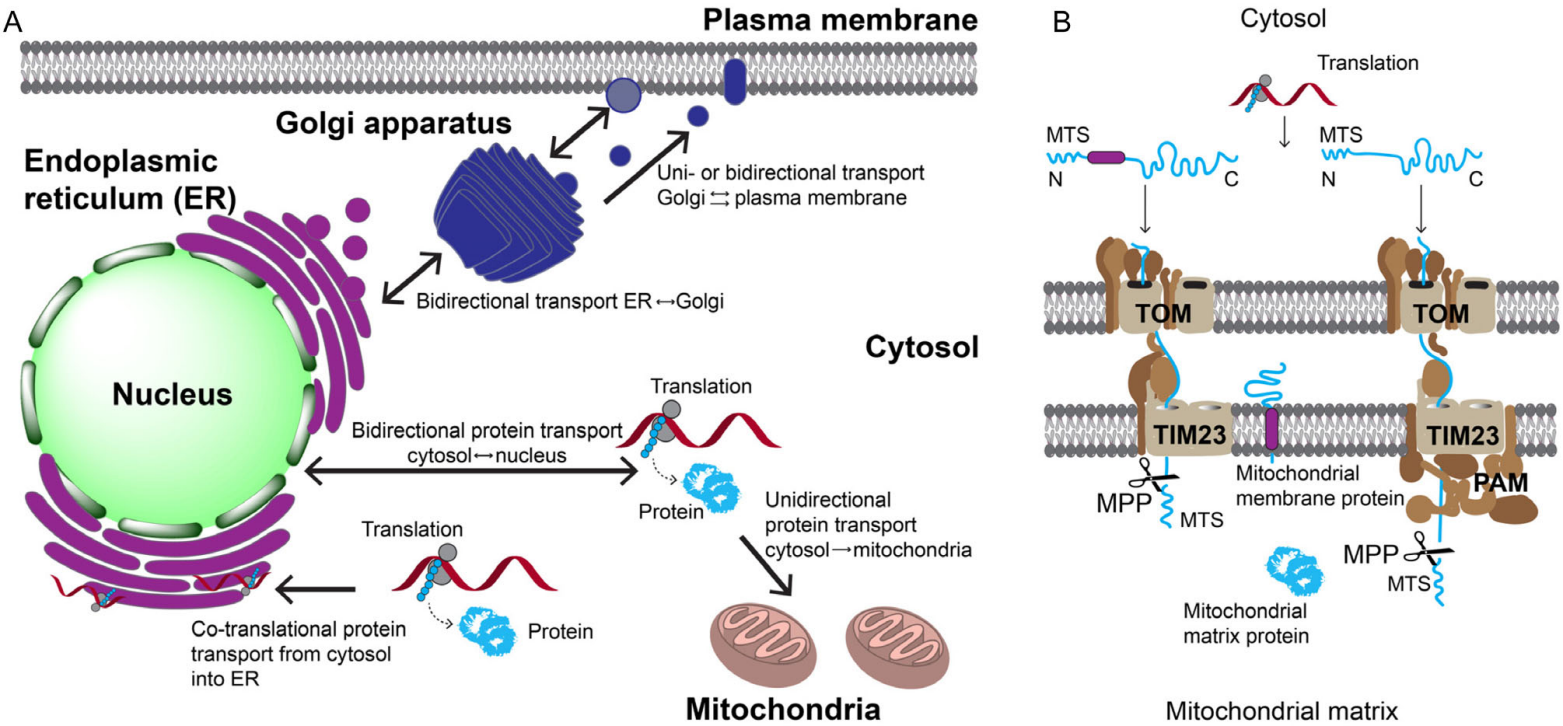
Schematic and simplified depiction of classical protein sorting pathways. A) All proteins except for 13 proteins encoded in the mitochondrial genome are translated in the cytosol. Nuclear proteins often shuttle back and forth between the nucleus and the cytosol. Transportation of proteins into the ER usually occurs in a cotranslational process when the growing nascent polypeptide chain is directly transferred into the ER lumen. Membrane and secretory proteins can shuttle between the ER and the Golgi and finally are transported to the membrane. Proteins internalized from the cell exterior via endosomes can be redirected to the Golgi. B) Many proteins to be localized in the mitochondria are transported posttranslationally via the TOM complex in the outer mitochondrial membrane and via the TIM23 complex in the inner mitochondrial membrane with final destinations in the inner mitochondrial membrane or the mitochondrial matrix. The N‐terminal mitochondrial targeting sequence (MTS) is cleaved off in the mitochondrial matrix via mitochondrial protein proteases (MPP). Image is adapted from Schmidt et al., 2010.^[^ ^37^ ^]^

With the exception of thirteen proteins encoded by the mitochondrial genome, which are transcribed and translated directly within mitochondria, the translation of all proteins occurs in the cytosol. Therefore, the cytosol serves as the central hub for subsequent intracellular protein distribution (**Figure** **1**A). Classically, only the nucleus was considered to support bidirectional exchange with the cytosol, mediated by import and export signals (via NLS and NES) and nuclear pore complexes.^[^ ^31^ ^]^ By contrast, organelles such as mitochondria or the ER were long viewed as terminal destinations: co‐ or posttranslationally imported proteins were thought to remain confined within these compartments or follow predefined export routes to the cell surface or secretory pathway, but not back to the cytosol or the nucleus (Figure 1A).^[^ ^32^ ^,^ ^33^ ^]^ Within the ER, many proteins undergo complex PTMs, such as glycosylation or lipidation, often in coordination with the Golgi apparatus, to become membrane‐bound receptors or secreted proteins. The molecular exchange between ER, Golgi, and plasma membrane occurs via a highly dynamic vesicular transport system.^[^ ^34^ ^]^ Conversely, proteins imported into mitochondria, whether localized to the membranes or the matrix, were long assumed to remain permanently compartmentalized (Figure 1B), subject only to degradation by mitochondrial proteases.^[^ ^35^ ^,^ ^36^ ^,^ ^37^ ^]^ The release of mitochondrial proteins into the cytosol is considered a potent cellular stress signal and is typically associated with the activation of the intrinsic apoptotic pathway.^[^ ^38^ ^,^ ^39^ ^]^ Thus, the retrograde transport of mitochondrial proteins into the cytosol or nucleus normally requires deliberate permeabilization of mitochondrial membranes.^[^ ^40^ ^]^

**Figure 2:**
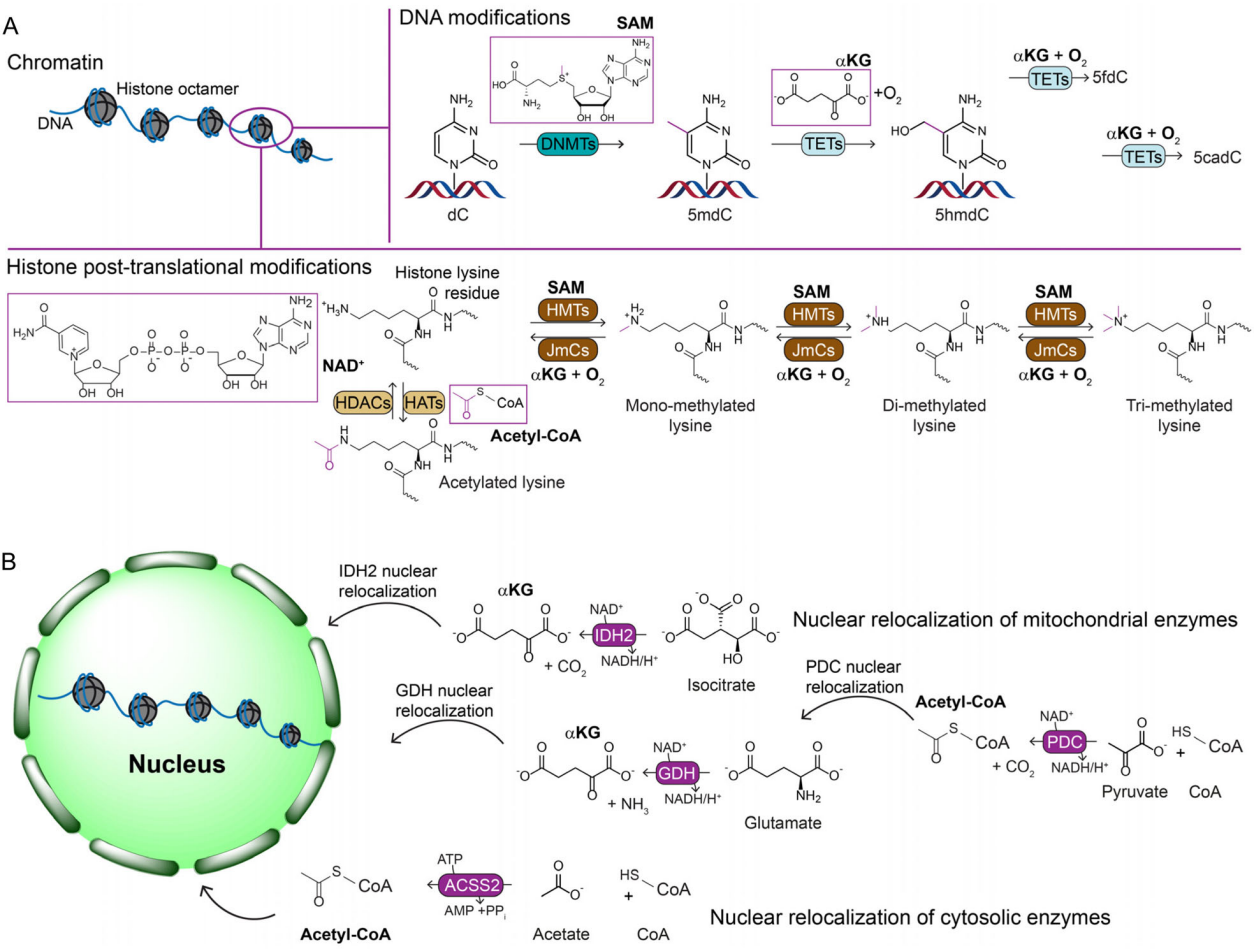
Metabolite requirements in the nucleus and nuclear moonlighting of metabolic enzymes. A) In eukaryotic cells, DNA is wrapped around an octamer of histone proteins to form the basic chromatin structure. Both DNA and histones undergo extensive chemical (epigenetic) modifications, which require specific metabolites as substrates or cofactors. Shown here are examples for methylation and acetylation, though many other modifications exist. DNA methylation, catalyzed by DNA methyltransferases (DNMTs), and lysine methylation by histone methyltransferases (HMTs) require SAM. DNA and histone demethylation, mediated by TET enzymes and JmjC‐domain‐containing histone demethylases, require *α*KG and oxygen. Acetylation reactions depend on acetyl‐CoA, while deacetylation by sirtuins requires NAD^+^. B) To supply these metabolites locally, several metabolic enzymes have been shown to translocate to the nucleus. These include normally mitochondrial *α*KG‐producing enzymes such as IDH2 and GDH, as well as the PDC for acetyl‐CoA production. Additionally, the cytosolic enzyme ACSS2 can relocate to the nucleus to provide acetyl‐CoA.^[^ ^43^ ^,^ ^47^ ^,^ ^56^ ^,^ ^58^ ^]^

However, in the past decade, accumulating evidence has shown that the cellular proteome is far more dynamic than previously appreciated.^[^ ^41^ ^]^ Increasing evidence shows that protein localization is context‐dependent and reversible, particularly during developmental transitions and cellular differentiation.^[^ ^42^, ^43^, ^44^, ^45^ ^]^ Many metabolic enzymes previously regarded as strictly mitochondrial can undergo regulated nuclear relocalization, providing local sources of metabolites required for epigenetic regulation.^[^ ^45^, ^46^, ^47^, ^48^, ^49^ ^]^ In the nucleus, metabolites such as S‐adenosylmethionine (SAM), acetyl‐CoA, *α*‐ketoglutarate (*α*KG), and nicotinamide adenine dinucleotide (NAD^+^) serve as cofactors for DNA and histone modifications (**Figure** **2**A) that control chromatin accessibility and gene expression. These modifications are dynamic, can be propagated during cell division but also remodeled during differentiation and other processes, and therefore create a high local demand of metabolites within the nucleus.^[^ ^50^, ^51^, ^52^, ^53^, ^54^, ^55^ ^]^ Although small metabolites can in principle diffuse through nuclear pores, direct nuclear localization of metabolic enzymes ensures on‐site metabolite supply for chromatin remodeling (Figure 2B).^[^ ^42^, ^43^, ^44^ ^,^ ^47^ ^,^ ^56^ ^,^ ^57^ ^]^ Prominent examples include the pyruvate dehydrogenase complex (PDC), which was discovered in the nucleus of cancer cell lines, where it produces acetyl‐CoA for histone acetylation in a cell‐cycle‐dependent manner to promote transcription of genes required for proliferation.^[^ ^47^ ^]^ Similar nuclear localization of PDC and proximal TCA enzymes has been observed during the maternal‐to‐zygotic transition in mouse embryos, where they supply acetyl‐CoA and *α*KG for epigenetic reprograming. By contrast, distal TCA enzymes remain mitochondrial and return to exclusive mitochondrial localization in later development stages.^[^ ^43^ ^]^ Nuclear relocalization of metabolic enzymes also occurs during neuronal differentiation and is essential for maintaining neuronal function. During neuronal differentiation and maturation, acetyl‐CoA for histone acetylation is not provided by nuclear PDC, but by a nuclear variant of acetyl‐CoA synthetase (ACSS2), which is typically cytosolic.^[^ ^57^ ^,^ ^58^ ^]^ Attenuation of nuclear ACSS2 in hippocampal neurons impairs the formation of long‐term spatial memory.^[^ ^58^ ^]^ In addition, our own work has shown that in adult murine neurons, *α*KG is provided by a nuclear variant of glutamate dehydrogenase (*GLUD1*/GDH) via interaction with ten‐eleven translocation methylcytosine dioxygenase 3 (TET3), which catalyzes the oxidation of 5‐methylcytosine (5mdC) to 5‐hydroxmethylcytosine.^[^ ^56^ ^,^ ^59^ ^]^ Although *GLUD1* contains a MTS, it is directly transported into the nucleus after translation, unlike the PDC, which translocates from mitochondria.

To date, these studies on nuclear relocalization of metabolic enzymes have been conducted primarily in cell culture and mouse models, particularly in the context of differentiation and neuronal maturation. However, emerging clinical evidence suggests that these mechanisms also play a role in human disease. For instance, a nuclear variant of hexokinase 2 has been shown to enhance stemness and tumorigenic potential in leukemic stem cells.^[^ ^42^ ^]^ Given the distinct metabolic signatures and vulnerabilities of tumor cells,^[^ ^60^ ^]^ the systematic and spatially resolved analysis of the nuclear relocalization of metabolic enzymes from the cytosol and mitochondria, and the resulting metabolic network formed within the nucleus, represents a promising approach for identifying novel biomarkers and therapeutic targets. Achieving this requires comprehensive and robust subcellular‐resolved protein profiling methodologies.

## 3 Imaging‐ and Mass Spectrometry–Based Spatial Protein Profiling

The analysis of subcellular protein localization has traditionally relied on fluorescence‐based microscopy, which remains among the most widely used methods.^[^ ^61^ ^]^ This approach requires specific labeling of the target protein with a fluorophore. Labeling can be achieved by fusing the protein to a fluorescent reporter such as GFP, by using fluorophore‐conjugated antibodies, or via small peptide tags such as the HALO tag, which can be selectively labeled using ligand‐based probes.^[^ ^62^, ^63^, ^64^, ^65^ ^]^ Modern high‐resolution microscopes enable precise localization of individual proteins within cells, as well as the analysis of their distribution across cell populations. Multiplexing approaches further allow simultaneous detection of multiple proteins, such as compartment‐specific markers or potential interaction partners. Subcellular localization is frequently validated using a complementary biochemical approach, in which cellular compartments are isolated, and the protein of interest is detected by immunoblotting alongside a compartment‐specific marker.

A major advantage of microscopy‐based methods is that they preserve cellular integrity, allowing direct assessment of both intra‐ and intercellular differences in localization. However, their greatest limitation lies in their targeted nature: these methods require prior selection of the proteins to be studied and are therefore unsuitable for comprehensive, unbiased proteomic analysis and identification of mechanistic networks. Unexpected localization patterns, such as the presence of metabolic enzymes in the nucleus or global relocalization of protein networks, which could provide insight into regulatory mechanisms, are typically missed. In contrast, MS‐based proteomic profiling enables a comprehensive analysis of the cellular proteome without the need for preselection of target proteins, which is the key advantage of this approach. When multiple subcellular compartments are analyzed and compared in parallel, this approach is referred to as spatial proteomics or organelle profiling. Depending on the specific methodology, these approaches can provide not only information on protein localization but also insights into localization dynamics and potential interactions.^[^ ^66^ ^,^ ^67^ ^]^

In addition to capturing the spatial distribution of the proteome in an unbiased manner, spatial proteomics offers an additional advantage over targeted microscopy‐ or antibody‐based techniques in general: quality control and normalization can be derived directly from the acquired data. This makes it possible to identify false positives or false negatives with greater reliability.^[^ ^68^, ^69^, ^70^ ^]^ In targeted microscopy‐based assays, for example, when using fluorophore‐conjugated antibodies against endogenously expressed proteins, false‐positive signals can arise from cross‐reactivity with other proteins or PTMs. To avoid such artifacts, antibodies must be validated under the exact same experimental conditions, ideally using knockout cell lines. However, this is not always feasible and generally requires additional resources. The limited specificity of many antibodies remains a well‐documented challenge in biomedical research, with potentially serious consequences, especially when therapeutic strategies are based on false‐positive findings.^[^ ^71^ ^,^ ^72^ ^]^ Further concerns arise in the context of immunoblotting, where signal bands can now be manipulated within seconds using generative AI tools, making intentional falsification increasingly difficult to detect. Omics‐based datasets are substantially less prone to these issues and are therefore better suited for robust and reproducible analyses. While deliberate data manipulation cannot be ruled out entirely, the inherent complexity of such datasets makes targeted falsification considerably more difficult. In bottom‐up proteomics workflows, for example, proteins are enzymatically digested into peptides, which are subsequently analyzed by MS. In the first measurement (MS1), the mass‐to‐charge ratio (*m/z*) and intensity of the intact peptides are recorded. Systematically, a subset of these peptides is then fragmented in a collision cell. The resulting fragments are analyzed in a second measurement (MS2) based on their *m/z* ratio. The combined MS1 and MS2 data enable unambiguous peptide identification and quantification based on signal intensity.^[^ ^73^ ^]^ The total intensity of a given protein is then calculated from the cumulative intensity of its constituent peptides, making targeted post hoc manipulation highly challenging.

Standardized normalization procedures are essential to ensure valid comparisons between experimental conditions (e.g., treated vs. nontreated cells) and to minimize the influence of technical variation during sample preparation and data acquisition. In targeted assays, which focus on a specific protein of interest, normalization is typically achieved using so‐called “housekeeper” proteins, which are reference proteins with expected stable expression across conditions. Common examples include structural proteins such as β‐tubulin or glycolytic enzymes such as GAPDH.^[^ ^74^ ^,^ ^75^ ^]^ However, under conditions of extensive cellular remodeling, expression of these reference proteins may also fluctuate, thereby compromising their suitability for normalization. Ideally, multiple housekeepers should be used in parallel, although this is rarely implemented in practice.

Proteomic approaches, in contrast, quantify the entire set of detectable proteins, allowing normalization to be based on the global dataset. This substantially improves data quality and statistical power. In organelle profiling, localization data are not limited to the protein of interest but encompass all quantified proteins. If one protein exhibits altered localization, the entire dataset can be leveraged to assess plausibility – for example, to distinguish specific relocalization events from global effects or technical artifacts introduced during sample preparation or analysis.^[^ ^76^ ^]^

Compared to conventional global proteomics or NGS‐based techniques, spatial proteomics requires considerably greater experimental and analytical effort. Only recent technological advances, particularly AI‐assisted tools for data processing and interpretation, have begun to mitigate these challenges and enabled broader application of spatial proteomic strategies. Ultimately, the choice of methodology depends heavily on the specific research question, and relevant considerations include the cellular compartments of interest, the required spatial resolution, scalability, and the available technical infrastructure. Since compartment‐specific separation of proteins cannot be achieved within the mass spectrometer itself, cellular compartments must be isolated or labeled prior to the MS‐analysis. This can be accomplished through subcellular fractionation, labeling‐based strategies, or microscopy‐guided segmentation. In practice, many modern workflows integrate complementary techniques, for example, combining biochemical enrichment with spatial labeling or imaging‐based validation. To provide a systematic overview, we maintain these three conceptual categories in the following sections, while cross‐referencing the other approaches where appropriate.

### 3.1 Subcellular Fractionation

**Figure 3:**
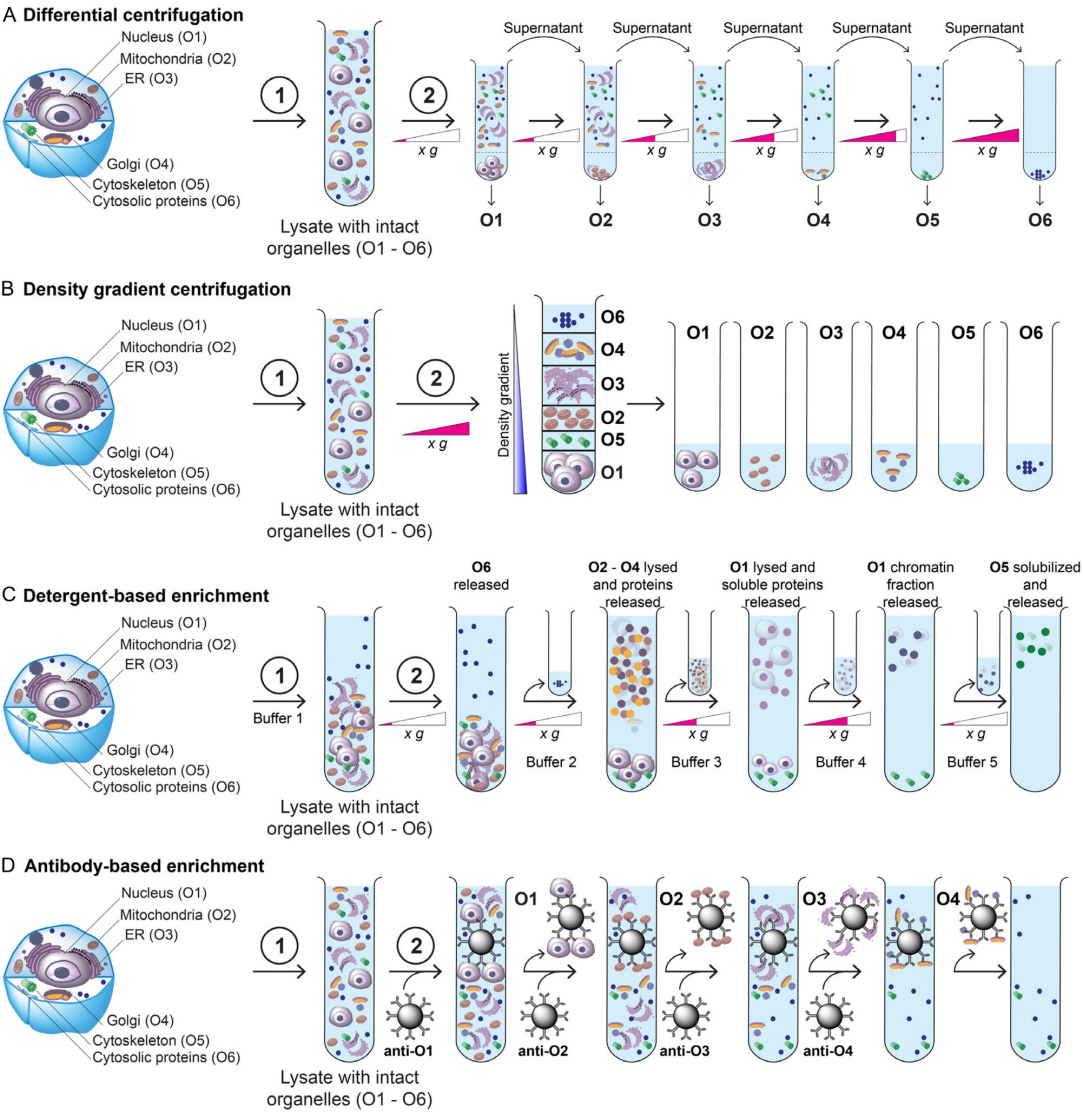
Common methods for cellular fractionation to isolate organelles for downstream analysis. Step 1 involves cell lysis to permeabilize the plasma membrane and release intact organelles. A) Differential centrifugation: Following lysis, samples are subjected to sequential centrifugation at increasing speeds, pelleting organelles according to their sedimentation rates. B) Density gradient centrifugation: Lysates are layered onto a density gradient (e.g., sucrose) and centrifuged, allowing organelles to migrate to their buoyant density, forming distinct bands for collection. C) Detergent‐based enrichment: Following lysis, proteins are sequentially extracted based on solubility. Those most readily solubilized are recovered first, while less soluble proteins are extracted in later steps. D) Antibody‐based enrichment: After lysis, antibodies (often bead‐coupled) targeting organelle surface proteins are used to sequentially capture and isolate specific organelles from the same sample.

In subcellular fractionation, cells are lysed using either mild detergents or mechanical methods, resulting in selective disruption of the plasma membrane while largely preserving the integrity of intracellular organelles (**Figure** **3**). The subsequent separation of organelles relies on their distinct physicochemical properties, such as differences in solubility, density, or size. Organelles can thus be isolated by centrifugation‐based techniques or sequential extraction.^[^ ^61^ ^,^ ^76^, ^77^, ^78^, ^79^, ^80^ ^]^ In systems that do not rely on human primary cells, it is also possible to genetically engineer cells to express compartment‐specific surface antigens on selected organelles. These can then be selectively enriched using high‐affinity antibodies.^[^ ^30^ ^]^ Because intracellular protein metabolism from synthesis to degradation is relatively slow, and the diffusion of large proteins is limited within cells, the subcellular distribution of proteins remains largely stable throughout the fractionation procedure.

#### 3.1.1 Centrifugation‐Based Methods

Centrifugation‐based techniques exploit the distinct physical properties of cellular organelles, particularly their sedimentation velocity and density, to enable the separation and analysis of subcellular proteins.

##### 3.1.1.1 Differential Centrifugation

Differential centrifugation separates organelles based on their varying sedimentation rates in aqueous solution (Figure 3A). Following cell lysis, typically achieved using a mild detergent that selectively permeabilizes the plasma membrane while releasing cytosolic proteins and preserving intact organelles, the lysate is subjected to a series of centrifugation steps at increasing centrifugal force. After each spin, the supernatant is carefully removed and further centrifuged in the next step. The resulting sediment fractions (pellets) are lysed under harsh conditions to extract all proteins, including membrane‐associated components, for downstream analysis.^[^ ^76^ ^,^ ^79^ ^,^ ^81^ ^,^ ^82^ ^]^ The required centrifugal speeds and resolution achieved depend heavily on the protocol and cell type used. Typical centrifugation steps involve sequential sedimentation of the nucleus, followed by mitochondria, then the permeabilized plasma membrane and ER. Endosomes/lysosomes and the Golgi apparatus require higher speeds, while protein complexes can be separated from smaller soluble cytoplasmic proteins using ultracentrifugation at very high speeds.^[^ ^81^ ^,82]^ The proteomes of additional organelles, such as exosomes or stress granules, have also been successfully isolated and characterized using differential centrifugation.^[^ ^83^ ^,^ ^84^ ^]^

##### 3.1.1.2 Density Gradient Centrifugation

Density gradient centrifugation is another centrifugation‐based method that separates organelles according to their buoyant density within a preformed gradient, typically generated using sucrose or Percoll (Figure 3B).^[^ ^79^ ^,^ ^80^ ^,^ ^85^ ^]^ The supernatant containing intact organelles is layered onto the top of the gradient and centrifuged at very high speeds (>100 000 × *g*). Organelles migrate through the gradient and accumulate at the point where their density matches that of the surrounding medium (the isopycnic zone). The resulting fractions form distinct bands that can be selectively extracted and analyzed. Depending on the centrifugation time, separation can be based purely on density (for long runs > 24 h) or additionally influenced by the sedimentation coefficient, which incorporates size and shape (for shorter runs).^[^ ^86^ ^]^ This technique has been successfully applied to isolate various organelles, including mitochondria, lysosomes, and Golgi vesicles.^[^ ^85^ ^,^ ^87^ ^]^

A major advantage of both centrifugation‐based approaches is their capacity for high‐resolution separation of organellar fractions while typically preserving organelle functionality. This allows not only MS‐based proteomic analyses but also compartment‐specific functional assays, such as measurements of mitochondrial enzyme activity.^[^ ^88^ ^]^ However, a key limitation of these methods is the relatively high amount of starting material required to ensure reliable fractionation. When working with small sample sizes, the risk of sample loss or cross‐contamination increases substantially. Additionally, these methods require specialized equipment (e.g., ultracentrifuges), and the handling of gradients demands experience and precision.

#### 3.1.2 Detergent‐Based Methods

Membrane composition and protein content vary significantly between organelles, leading to differences in solubility under specific buffer conditions that differ in detergent type, pH, and ionic strength. Insoluble components can be separated from solubilized proteins by centrifugation. However, detergent‐based protocols cannot achieve complete separation of all cellular compartments at high resolution. These methods are commonly used to distinguish cytoplasmic, nuclear, and other membrane‐enclosed fractions, including mitochondria.^[^ ^89^, ^90^, ^91^ ^]^ The isolation strategy typically proceeds stepwise, from readily to poorly soluble components (Figure 3C). Initially, the plasma membrane is selectively permeabilized using a mild detergent such as digitonin, which interacts preferentially with cholesterol‐rich membranes. As a result, cytoplasmic proteins are released, while organelle membranes, stabilized by specific lipids such as cardiolipin, remain largely intact.^[^ ^79^ ^]^ Following removal of the cytoplasmic fraction, the remaining cell pellet can be extracted with a stronger nonionic detergent such as Triton X‐100, which solubilizes membrane‐associated proteins from organelles other than the nucleus. For the extraction of nuclear proteins, particularly those associated with the nuclear matrix or chromatin, harsher lysis conditions are required. These typically involve ionic detergents such as sodium deoxycholate or sodium dodecyl sulfate (SDS), which effectively disrupt stable protein complexes within the nucleus. Often, cytoskeletal proteins are the last ones to be solubilized. While the limited resolution between fractions and the frequent denaturation of organelles during sample preparation represent clear disadvantages compared to centrifugation‐based methods,^[^ ^77^ ^]^ this approach offers advantages in terms of user‐friendliness, both with respect to sample input requirements and ease of sample processing.^[^ ^92^ ^]^

#### 3.1.3 Antibody‐Based Methods

An efficient approach for isolating even small or physicochemically challenging organelles in high purity from limited sample amounts is immunoprecipitation (IP) targeting an organelle‐specific surface protein (Figure 3D). In this method, cells are first permeabilized and then sequentially incubated with antibodies against characteristic surface proteins of the target organelle or characteristic components of protein complexes—for example, against the TOMM20 complex in the outer mitochondrial membrane or components of the nuclear pore complex for nucleus isolation.^[^ ^30^ ^]^ The antibodies are coupled to carrier materials such as magnetic beads, enabling the intact “fishing out” of the organelles. The isolated organelles are then lysed to release their proteins for downstream analysis.

A key advantage of this method, beyond its suitability for small sample volumes and organelles that are difficult to separate, is the short preparation time. Moreover, because of the purification speed, the approach can be applied not only to proteins but also to organelle‐specific metabolites, provided they are enclosed by a highly selective membrane, as in mitochondria.^[^ ^93^ ^,^ ^94^ ^]^ Coupling antibodies to magnetic beads or similar carriers makes the process in principle amenable to automation. For very small cell numbers, sample integrity can be further preserved by minimizing transfer steps: the unbound fraction remains in the reaction vessel, while only the beads with bound organelles are transferred (Figure 3D). A limitation of antibody‐based pulldown approaches is the high requirement for antibody quality: antibodies must be highly specific, IP‐compatible, and capable of enriching the target organelle with sufficient efficiency to avoid cross‐contamination with other cellular compartments. Consequently, many published organellar profiling studies employing IP‐based separation do not use antibodies against endogenous surface markers. Instead, the target organelle is engineered in genetically modified cells to express an artificial epitope, such as GFP, HA‐tag, or another fusion tag, on its surface.^[^ ^94^ ^,^ ^95^ ^]^ Purification is then carried out with antibodies against this artificial epitope, substantially improving specificity and yield. Using this strategy, highly resolved organelle maps of cells before and after viral infection have, for example, already been successfully generated.^[^ ^30^ ^]^ However, this approach requires extensive genetic cell engineering and is therefore generally not directly transferable to primary human cells, such as those obtained from clinical samples.

### 3.2 Labeling‐Based Approaches

Protein translocation is often a dynamic and context‐dependent process, influenced by the local protein environment and cellular state. These transient or condition‐specific localization changes can be difficult to capture by subcellular fractionation, which provides a comprehensive global view but is limited in sensitivity to subtle or short‐lived translocation events of specific proteins. When specific translocation events and subcellular contexts are of interest, proximity labeling (PL) approaches, originally developed to study protein–protein interactions, have emerged as powerful tools for probing the spatial proteome and uncovering translocation‐dependent protein networks within their native cellular context. Conceptually, PL methods can be classed as enzyme‐driven, photocatalytic, or microscopy‐guided photolabeling, that vary in targeting and activation strategy.

#### 3.2.1 Enzyme‐driven PL

In enzyme‐driven PL, the target protein is typically genetically fused to an enzyme that, upon addition of a suitable substrate such as biotin, generates reactive intermediates capable of covalently tagging nearby proteins. Because biotin occurs at only very low concentrations in most cell types, the specifically tagged proteins can be selectively enriched via streptavidin beads and subsequently identified by MS, while unlabeled proteins are washed away.^[^ ^96^, ^97^, ^98^, ^99^ ^]^ This selective labeling allows all relevant cells to be processed together (pooling), which, by increasing the total sample volume, greatly enhances sensitivity.^[^ ^97^ ^]^ Thus, detection of both strong and transient interactions as well as tracking of protein translocations is possible and has been demonstrated in varied cell types and species to map the proteome composition of diverse organelles.^[^ ^99^, ^100^, ^101^, ^102^ ^]^ The first enzyme‐driven PL system, BioID, employs a mutant E. coli biotin ligase (BirA) fused to a bait protein to catalyze biotin‐dependent covalent tagging of nearby interactors.^[^ ^103^ ^]^ Although broadly applicable, early BioID variants showed limited catalytic efficiency and temporal control. Subsequent derivatives, for example, BioID2, TurboID, and miniTurbo, introduced improved biotin sensitivity, tunability, and faster labeling kinetics, greatly enhancing experimental flexibility.^[^ ^97^ ^,^ ^104^ ^,^ ^105^ ^]^

Split‐PL strategies, in which the enzyme is divided into two fragments that reconstitute only upon colocalization, reduce background labeling and allow detection of transient or contact‐dependent interactions. Examples include ContactID (split‐BioID) and split‐TurboID, both widely used for profiling membrane and cell–cell interfaces.^[^ ^97^ ^,^ ^106^ ^]^

In peroxidase‐based PL, addition of H_2_O_2_ activates horseradish peroxidase (HRP) or engineered ascorbate peroxidase (APEX) to generate short‐lived biotin‐phenoxyl radicals from previously added biotin‐phenol that label neighboring proteins. HRP is restricted to extracellular or luminal environments, whereas APEX and its improved variant APEX2 enable intracellular labeling with high temporal resolution.^[^ ^107^, ^108^, ^109^ ^]^ Recent orthogonal PL systems, such as TransitID, which combines APEX2 and TurboID sequentially to capture protein movement between compartments, enable multiplexed mapping of dynamic trafficking pathways.^[^ ^110^ ^]^

As an alternative to genetic modification of the protein of interest, antibody‐based enzyme‐driven PL can be employed. In this approach, a primary antibody specific to the target antigen and conjugated to an labeling enzyme such as TurboID directs the deposition of biotin or another reactive label onto neighboring proteins.^[^ ^111^ ^]^ When high‐quality antibodies are available, this strategy is highly versatile, as it can be applied to primary samples and enables the simultaneous labeling of multiple targets in parallel.

#### 3.2.2 Photo‐PL

Photo‐PL (PPL) represents a rapidly evolving class of techniques that use light to generate reactive intermediates for covalent tagging of nearby proteins with high spatial and temporal precision. Unlike enzyme‐driven methods, which rely on continuous enzymatic activity, PPL allows instantaneous activation and strict spatiotemporal control of labeling events, making it particularly suited for studying dynamic or compartmentalized processes.^[^ ^112^, ^113^, ^114^, ^115^ ^]^

Two main PPL strategies have emerged. Microscopy‐guided photoactivation employs photoactivatable reagents such as benzophenone–biotin conjugates, which are locally activated by focused light to crosslink proteins within a predefined subcellular region.^[^ ^116^ ^]^ Microscopy‐guided photoactivation enables labeling within a specific optical region of interest but is typically limited to fixed or optically accessible samples. This approach is described in more detail in the following imaging‐based segmentation section.

In contrast, photocatalytic PL does not rely on microscopic targeting but instead uses light‐activated photosensitizers bound to the target protein or region of interest to catalyze the formation of reactive intermediates that diffuse over nanometer scales to tag proximal proteins.^[^ ^112^, ^113^, ^114^, ^115^ ^]^ Iridium‐based systems such as µMap use engineered split inteins to introduce iridium photocatalysts into defined cellular compartments, where they activate diazirine warheads via Dexter energy transfer, generating reactive carbenes that crosslink neighboring proteins within an ≈10 nm radius.^[^ ^117^ ^]^ This enables nanoscale mapping of protein–protein interactions and local proteomes in live cells.^[^ ^118^ ^]^ The concept of µMap has been successfully applied to reveal mutation‐ and drug‐induced remodeling of nuclear interactomes, providing powerful insights into epigenetic signaling and cancer‐associated network dynamics.

Red‐shifted variants, such as µMap‐Red, extend activation wavelengths into the visible spectrum, enhancing tissue penetration and moving the technology toward in vivo compatibility.^[^ ^119^ ^]^ Multiscale platforms such as MultiMap, which works with Eosin Y, a photocatalyst activating different types of photoactivatable reagents, enable adaptable labeling radii.^[^ ^120^ ^]^ Both, µMap‐Red and MultiMap employ photocatalyst‐conjugated antibodies, which enables labeling without prior genetic engineering. Beyond these platforms, flavin‐based photocatalytic platforms were reported to offer genetically encodable photosensitizers that function under milder conditions and allow intracellular or organelle‐specific targeting.^[^ ^121^ ^]^

Overall, PPL approaches provide an alternative to enzyme‐based PL, combining high temporal resolution, chemical precision, and minimal perturbation of the native proteome. As the technology matures, integration of PPL with complementary spatial proteomics and high‐content imaging techniques promises to uncover transient and context‐specific protein interactions inaccessible to conventional methods.

### 3.3 Imaging‐Based Segmentation

Spatial proteomics was named “Method of the Year” by *Nature Methods* in 2024. This designation primarily referred to approaches enabling spatially resolved proteome analysis in tissue sections. A central contribution in this field comes from mass spectrometry imaging (MSI) technologies, which combine region‐specific measurements with mass spectrometric analysis.^[^ ^122^ ^,^ ^123^ ^]^ The aim of these approaches is to unite the strengths of both worlds: integrating the high‐resolution spatial information on protein localization in intact tissues or cells provided by imaging with the high multiplexing capacity, accurate protein identification, and quantitative precision achievable by MS.

#### 3.3.1 Targeted Approaches

MSI methods include techniques that, like conventional imaging approaches, rely on antibody‐based labeling of individual proteins, most notably imaging mass cytometry (IMC) and multiplexed ion beam imaging (MIBI).^[^ ^124^ ^,^ ^125^ ^]^ Both use antibodies conjugated to stable metal isotopes but differ in their ionization strategies. IMC employs a laser for point‐by‐point ablation of the tissue, whereas MIBI uses a focused primary ion beam to release secondary ions from the sample. In both cases, the characteristic isotope signals are detected simultaneously. IMC achieves a moderate spatial resolution of ≈1 µm, whereas MIBI offers a substantially higher resolution of around 200 nm but requires more elaborate sample preparation and measurement protocols.^[^ ^124^, ^125^, ^126^ ^]^ In both methods, the high mass resolution of modern mass spectrometers enables precise separation of isotope signals, thereby avoiding the spectral overlap commonly encountered in fluorescence‐based imaging techniques.

A major limitation of IMC and MIBI is their target dependency. Despite employing MS as a readout, they are restricted to preselected proteins labeled with specific antibodies and, unlike unbiased LC–MS/MS approaches, cannot characterize the entire proteome. Moreover, in currently established MSI workflows, only the isotope label of the antibody is detected via its *m/z* ratio; there is no independent sequence analysis of the target protein following fragmentation, as is standard in LC–MS/MS workflows. As a result, quantitative data, similar to conventional imaging, remain entirely dependent on antibody quality and the full potential of mass spectrometry for comprehensive and systematic protein identification is not fully realized.

#### 3.3.2 Untargeted Approaches

In addition to antibody‐based MSI techniques such as IMC and MIBI, there are also MSI–based imaging approaches that operate independently of antibodies, thereby enabling an unbiased analysis. Among the most established methods is matrix‐assisted laser desorption/ionization (MALDI) imaging, which integrates high‐resolution spatial information with comprehensive mass spectrometric characterization of the local proteome.^[^ ^127^, ^128^, ^129^ ^]^ In this approach, molecules are detected following laser‐induced ionization in the presence of an appropriate matrix. Depending on the mode, either selected regions of the tissue are specifically analyzed (*profiling mode*) or the tissue is systematically scanned in a raster pattern (*imaging mode*). Linking the mass spectrometric signals to the precise laser position coordinates enables spatially resolved visualization of the molecular distribution within the tissue.

Efficient ionization requires prior treatment of the sample with a suitable matrix. The choice of matrix depends on the class of molecules to be analyzed, for example, sinapinic acid for proteins or α‐cyano‐4‐hydroxycinnamic acid for peptides.^[^ ^130^ ^,^ ^131^ ^]^ MALDI imaging can be performed either in a *top‐down* approach, analyzing intact proteins, or in a *bottom‐up* approach, in which the tissue or cell layer is treated with a protease solution, typically trypsin, before matrix application, thereby digesting proteins into peptides.^[^ ^132^ ^]^

**Figure 4:**
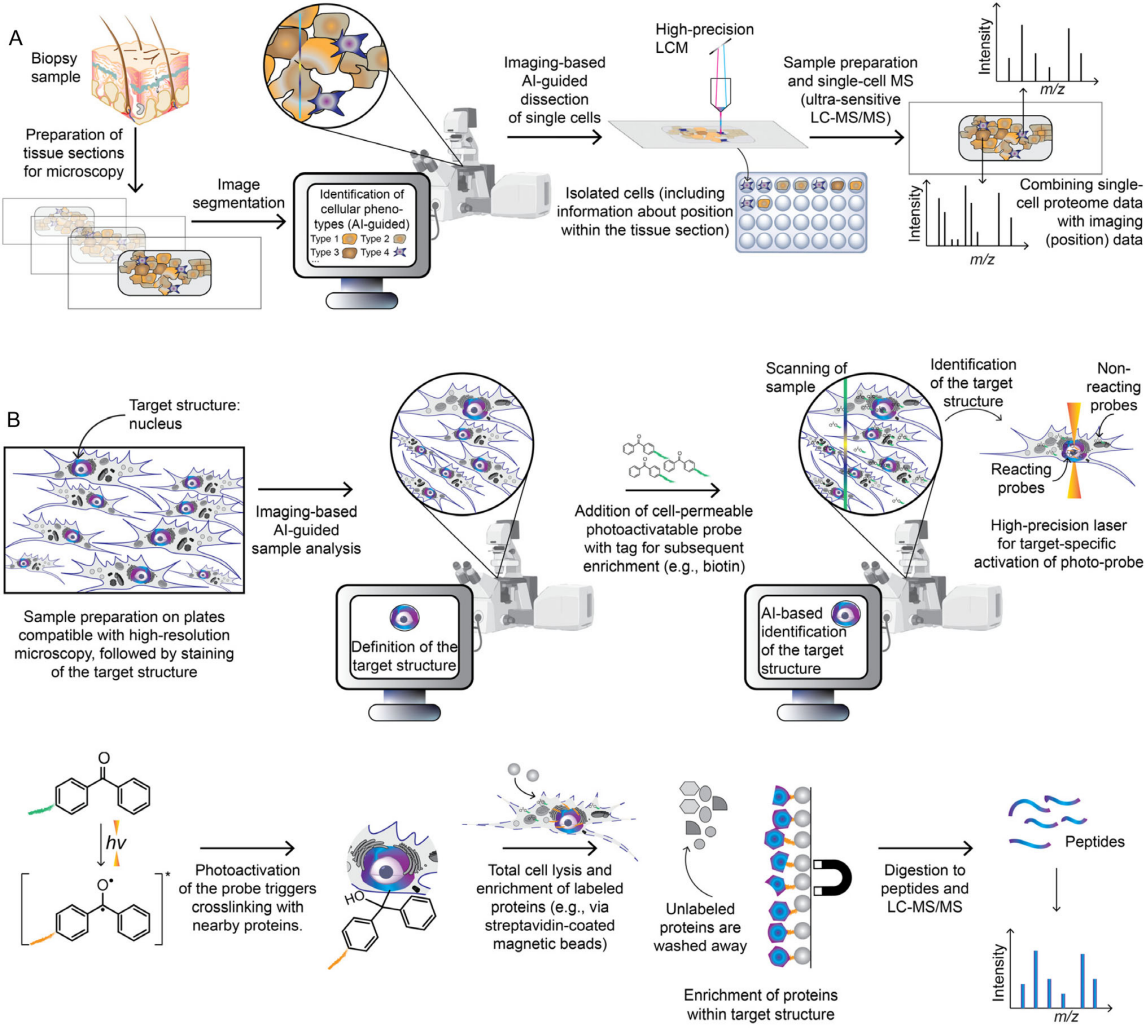
Schematic representation of imaging‐based segmentation spatial proteomics approaches. A) Representation of DVP workflow. Tissue sections from biopsy samples are imaged at high resolution, and individual cells are identified and classified using deep‐learning–based segmentation. Selected cells are isolated by high‐precision LCM, lysed, digested, and analyzed by ultrasensitive LC‐MS/MS. Spatial cell‐to‐proteome mapping enables detection of both cell‐type‐specific and location‐dependent proteomic differences, and the high imaging resolution permits direct correlation of morphological features with proteomic profiles. B) Microscopy‐guided photo‐labeling for spatial proteomics. Cells or tissue sections are first partially imaged to visualize the target structure, such as the nucleus, via a fluorescent signal or other marker. AI‐based image analysis generates a mask of this structure. A cell‐permeable, photoactivatable probe containing an enrichment tag (e.g., biotin) is then applied to the entire sample. The whole sample is subsequently reimaged, and the previously generated mask is applied. A high‐precision laser selectively activates the probe within the masked region, covalently labeling nearby proteins. Finally, the sample is lysed, and labeled proteins are enriched via the incorporated tag for downstream LC‐MS/MS analysis.

A recently developed, high‐resolution and highly sensitive form of MS‐based imaging is deep visual proteomics (DVP) (**Figure** **4**A).^[^ ^133^ ^]^ In this approach, tissue is first imaged at high microscopic resolution. Individual cells are then identified and automatically classified based on morphological features using deep learning–assisted image segmentation. Targeted isolation of these cells is achieved via high‐precision laser capture microdissection (LCM). AI‐driven segmentation enables standardized sample preparation, ensuring high reproducibility. The isolated cells are subsequently lysed, proteolytically digested, and subjected to ultrasensitive LC‐MS/MS analysis, allowing comprehensive characterization of their proteomes. Because each cell can be unambiguously assigned to its spatial position within the tissue, both cell type–specific differences and location‐dependent variations in the proteome of the same cell type can be resolved. The high microscopic resolution further enables direct correlation of morphological features with the associated proteomic data during subsequent bioinformatic analysis. These features make DVP a particularly powerful tool for studying solid tumors, where tumor development and progression are strongly influenced by the complex tumor microenvironment, as it allows protein‐level links to be drawn between the diverse cell types present.^[^ ^134^ ^,^ ^135^ ^]^ Although tumor tissue was the first application, the method is broadly applicable for the characterization of clinical tissue samples, especially since the individual steps can be largely automated given appropriate instrumentation.Currently, both MALDI imaging and DVP are limited in spatial resolution mostly to the cellular level, as neither laser desorption nor LCM can yet achieve targeted isolation of very small subcellular structures.^[^ ^127^ ^,^ ^129^ ^,^ ^133^ ^,^ ^135^ ^]^ While nuclei as the largest subcellular structure, have already been analyzed successfully, smaller organelles remain challenging. However, advances in optical resolution, combined with AI‐driven image analysis, are expected to enable the targeted investigation of smaller, morphologically well‐defined organelles with sufficient protein content, such as mitochondria, in the future.

#### 3.3.3 Photolabeling‐Based Imaging Approaches

While methods such as DVP and MALDI imaging enable cell type‐specific and spatially resolved proteomic analysis, they face technical limitations when it comes to directly isolating small or hard‐to‐access subcellular compartments. To overcome these challenges while retaining the strengths of imaging‐based approaches, microscopy‐guided photolabeling has become a powerful alternative in spatial proteomics because it affords high control over where and when labeling occurs (Figure 4B).^[^ ^116^ ^,^ ^136^, ^137^, ^138^ ^]^ Here, cells or tissue sections are first imaged at high resolution, and target structures, such as specific cell types or subcellular compartments, are identified using antibodies, fluorescent tags, or morphological criteria. AI‐based image analysis generates a digital “mask,” which can then be automatically applied to other image fields. The sample is subsequently incubated with a cell‐permeable photoactivatable reagent such as benzophenone,^[^ ^139^ ^]^ which is conjugated to an enrichment tag such as biotin. In the second round of imaging, which covers the entire sample, a high‐precision laser activates the reagent only within the predefined masked regions. After activation, the reagent reacts with surrounding biomolecules, in particular proteins, thereby labeling them. The labeled proteins can then be specifically enriched for subsequent MS‐analysis. An exemplary workflow is shown in Figure 4B, where a photoactivatable benzophenone reagent bearing a biotin moiety is used. Upon light activation of benzophenone using a focused laser within the predefined nuclear target region, photo‐crosslinking occurs between the reagent and the peptide backbones of nearby proteins, thereby covalently attaching the biotin label to proteins residing in that region. After labeling, cells are lysed under stringent conditions to release the total protein content. Biotinylated proteins are then selectively enriched using magnetic streptavidin beads, while unlabeled proteins, that is, those not being present within the illuminated nuclear region, are washed away and excluded from subsequent MS analysis. As for enzyme‐driven PL, this selective labeling allows all relevant cells to be pooled, which, by increasing the total sample volume, greatly enhances sensitivity. Furthermore, the predefined mask enables targeted acquisition of cell type‐specific subcellular proteomes within the intact cellular environment, even for small or difficult‐to‐access compartments. The precise spatial control of labeling produces a high signal‐to‐noise ratio and substantially reduces contamination from other compartments.

A limitation of the method is that only one target compartment can be labeled per experiment, meaning that the resulting data cannot be directly related to other cellular compartments of the same cells. This can be addressed by sequentially defining different compartments as masks in separate but comparable samples; however, the proteomic data will then come from different cells, limiting direct comparability. When only a single organelle is of interest, however, the imaging‐guided labeling approach offers a clear advantage: the target organelle's proteome can be isolated with high specificity while fully preserving cellular architecture.

## 4 MS Acquisition, Quantification Strategies and Bioinformatic Analysis for High‐resolution Profiling of the Subcellular Proteome

### 4.1 MS Acquisition

Regardless of the method used to isolate organelle‐specific proteomes, major technological advances in both modern mass spectrometers and the bioinformatic analysis of acquired spectra and resulting protein intensity distribution profiles have enabled the generation of organellar maps with unprecedented depth and resolution.^[^ ^76^ ^,^ ^77^ ^,^ ^80^ ^,^ ^81^ ^,^ ^140^ ^,^ ^141^ ^]^ State‐of‐the‐art instruments, such as the latest Orbitrap and timsTOF platforms, combine high sensitivity, excellent mass resolution, fast scan rates, and robust performance. They achieve sub‐ppm mass accuracies and resolving powers, defined as the *m/z* of the peak divided by the width of that peak at half of its intensity (full width at half maximum [FWHM]), exceeding 200000 FWHM, allowing precise analysis of even highly complex samples with dense chromatographic peak profiles. Improvements in ion transmission and focusing, as well as optimized ion sources, have increased sensitivity to the point where analyses can now be performed on only a few cells, and even at the single‐cell level.^[^ ^5^ ^]^ The introduction of ion mobility spectrometry, for example in the form of timsTOF‐PASEF or FAIMS, has added an orthogonal separation dimension that reduces interferences and significantly improves identification rates, particularly in complex samples.^[^ ^142^, ^143^, ^144^ ^]^

**Figure 5:**
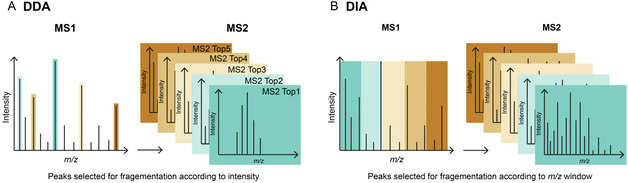
Principle of DDA and DIA. A) In DDA, during each MS1 cycle a fixed number of the most intense precursor peaks is selected, individually fragmented, and analyzed in the MS2. B) In DIA, all precursor ions within the full *m/z* range are sequentially fragmented in predefined *m/z* windows. Each window contains multiple precursors that are cofragmented, and the window is shifted stepwise until the entire *m/z* range is covered.

Acquisition strategies have also advanced considerably over the past decade. While data‐dependent acquisition (DDA) was once the sole standard for large‐scale proteomic analyses, data‐independent acquisition (DIA) has now become firmly established.^[^ ^70^ ^,^ ^145^ ^,^ ^146^ ^]^ In DDA, all peptides within a defined *m/z* range are first measured in an MS1 scan, and then the *n* most intense precursor ions per cycle are selected for fragmentation and subsequent MS2 analysis (“Top‐*n*” approach) (**Figure** **5**A). The number of precursors analyzed is directly limited by the instrument's scan speed. Because selection is stochastic, low‐abundance peptides may be detected in one run but not in another, even if present at similar levels, leading to reduced reproducibility between biological replicates.

DIA circumvents this limitation by fragmenting all precursor ions within consecutive *m/z* windows in each MS1 cycle, ensuring that even low‐abundance peptides are sampled (Figure 5B).^[^ ^145^ ^]^ However, this approach produces much more complex MS2 spectra, as each spectrum contains fragments from multiple precursor ions. Assignment therefore relies on spectral libraries, which have traditionally been generated from prior DDA runs but can now also be created in silico.^[^ ^147^ ^]^ The window width can be fixed or variable and must be optimized to cover the entire target *m/z* range (typically 400—1200 *m/z*) within a single cycle while keeping spectral complexity, and thus the computational demands of AI‐assisted analysis, at a manageable level.^[^ ^145^ ^]^

The combination of improved instrumentation, faster scan speeds, and more powerful analysis algorithms has made DIA a highly sensitive, reproducible, and near‐comprehensive method for proteomics. Today, tens of thousands of peptides can be identified and quantified in a single LC‐MS/MS run, even from highly limited starting material. These developments have been pivotal for generating high‐resolution organellar maps: while large organelles typically contain sufficient protein for deep analysis, smaller organelles yield much less material. Moreover, the variance between biological replicates after fractionation‐based sample preparation is often higher than in whole‐proteome analyses. Achieving comprehensive and reproducible peptide coverage within a single run is therefore critical for obtaining the necessary data depth and quality.

### 4.2 Quantification Strategies

Comprehensive analysis of the subcellular proteome is closely linked to the chosen quantification strategy. In most cases, the goal is not only to determine whether a given protein is present in a specific organelle, but also to assess its relative abundance, an aspect that is particularly critical for quality control. Quantitative data obtained via MS analysis enable the generation of protein distribution profiles across different organelles. Such profiles allow high‐resolution, organelle‐specific proteome analysis without requiring perfectly pure organelle isolations.^[^ ^76^ ^,^ ^77^ ^,^ ^80^ ^,^ ^82^ ^]^ Even with only partial separation of organelles, quantitative analysis of all fractions combined with comparison of protein distribution profile to an invariant reference profile and established organellar markers can yield high‐resolution localization profiles using multivariate statistical analysis and machine‐learning approaches.^[^ ^77^ ^]^ If a protein that is normally assigned to a particular organelle shows a high abundance in another, for example, in the case of mitochondrial proteins with nuclear “moonlighting” functions, distribution profiles can help distinguish between a genuine relocalization event and an artifact arising from sample preparation, measurement, or data analysis. For instance, if under certain conditions the majority of mitochondrial proteins remain predominantly localized in the mitochondria while only selected proteins are enriched in the nucleus, this suggests a specific biological effect. In contrast, if nearly all mitochondrial proteins exhibit increased abundance or even peak fractions in the nucleus, this more likely indicates insufficient separation of organelles during sample preparation or analysis.

**Figure 6:**
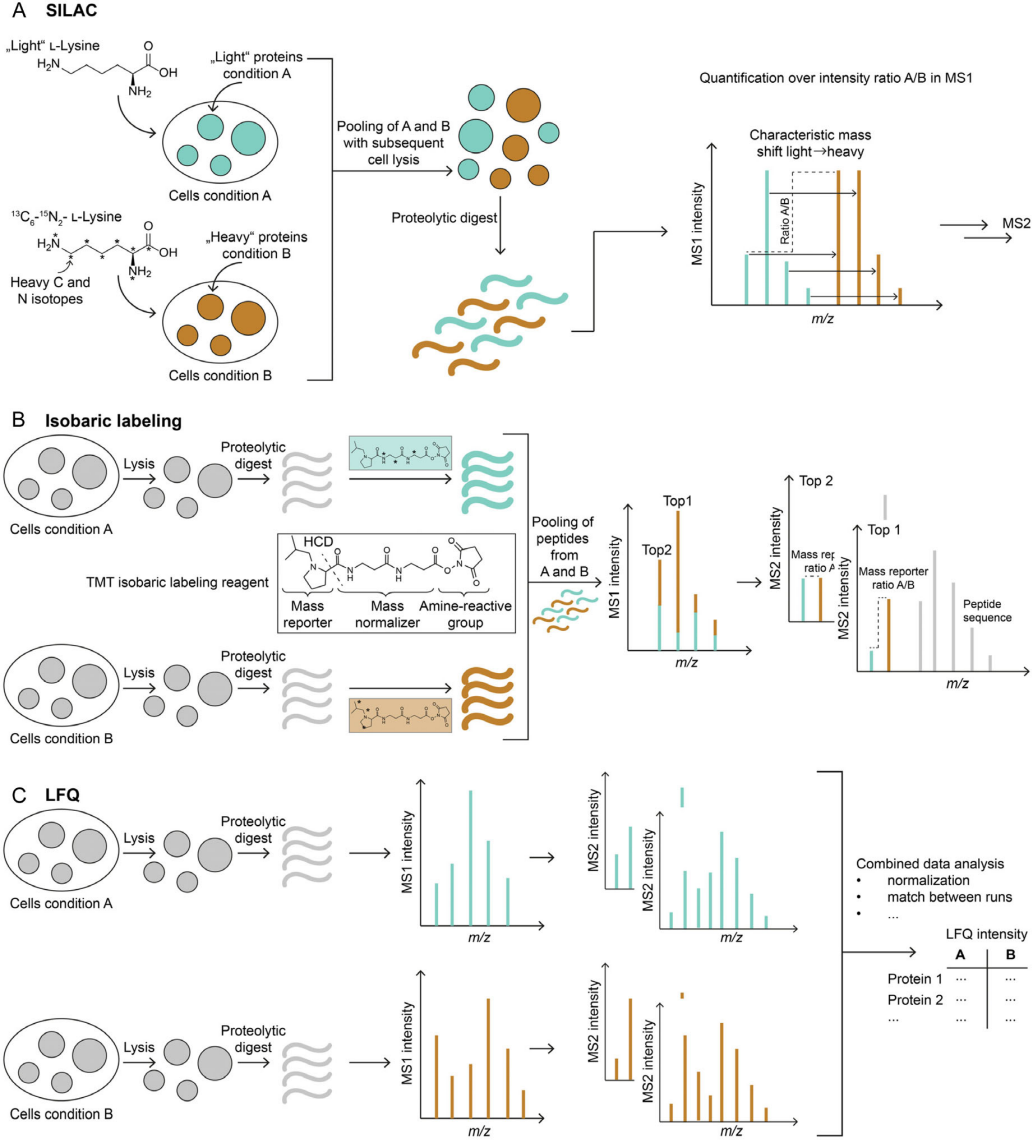
Principles of SILAC, isobaric labeling, and LFQ for quantitative proteomics. A) SILAC: Cells are metabolically labeled by incorporating heavy isotope–labeled amino acids in one condition (e.g., condition B), while the other (condition A) contains only light amino acids. This allows peptides from the two conditions to be unambiguously distinguished in the MS1 by a characteristic mass shift. After harvesting, cells from both conditions can be pooled, and all subsequent steps are performed jointly. Quantification is based on the MS1 intensity ratio of light to heavy peptide pairs with identical sequences. B) Isobaric labeling (e.g., TMT): Samples are processed separately until after proteolytic digestion, when peptides are labeled with chemically identical isobaric tags that differ only in their isotopic distribution. Labeled peptides are then pooled for joint MS analysis. In the MS1, peptides appear at the same mass; quantification occurs at the MS2 level, where high‐energy collision dissociation cleaves the tags to release mass reporters. The distinct reporter masses can be unambiguously assigned to each condition, and their intensity ratios provide quantitative information. C) LFQ: Samples are processed and measured separately. Quantification is performed during data analysis by aligning retention times, matching identical peptides across runs, and comparing their MS1 intensities between conditions.

The three most established quantification strategies for MS‐based proteomics data are stable isotope labeling by amino acids in cell culture (SILAC), isobaric labeling, and label‐free quantification (LFQ) (**Figure** **6**).^[^ ^68^ ^,^ ^148^ ^,^ ^149^ ^]^

#### 4.2.1 SILAC

This method is based on the metabolic incorporation of isotopically labeled amino acids into proteins during cell culture. Cells of the reference sample (e.g., sample A) are grown in medium containing only light amino acids, whereas cells of the comparison sample (e.g., sample B) are grown in medium in which one amino acid is present exclusively in a heavy isotope variant, such as ^13^C_6_‐^15^N_2‐_L‐lysine. This defined labeling allows peptides from the two samples to be unambiguously distinguished in the MS1 spectrum based on the known mass shift (Figure 6A).^[^ ^148^ ^,^ ^150^ ^]^ A key advantage is that samples can be combined immediately after cultivation, so that all subsequent steps from sample preparation to measurement are performed jointly. This minimizes technical variation and ensures maximal comparability. Quantification is based on the MS1 intensities of the corresponding peptides. Limitations include the restricted applicability to samples that cannot be cultured and metabolically labeled (e.g., human primary samples), the limited number of samples that can be compared simultaneously, and the increased complexity of MS1 spectra.

#### 4.2.2 Isobaric Labeling

The labeling with isobaric reagents (e.g., Tandem Mass Tags, TMT) addresses some of these limitations. In this approach, peptides are labeled after proteolytic digestion. Isobaric tags consist of a reactive group (e.g., amino‐reactive NHS ester), a mass normalizer, and a mass reporter.^[^ ^149^ ^,^ ^151^ ^]^ All tag variants have the same nominal mass but differ in the isotopic distribution between the normalizer and reporter regions. Consequently, labeled peptides of the same nature but from different samples appear as a single peak in MS1. As a result, they are cofragmented and are finally distinguished in MS2 based on the mass reporters (Figure 6B). Quantification is thus performed using the reporter ion intensities. Advantages include higher multiplexing capacity and no increase in MS1 complexity. However, isobaric labeling is less compatible with standard DIA workflows, as the unambiguous assignment of reporters to specific precursors is more challenging in this acquisition mode.

#### 4.2.3 LFQ

The most flexible and cost‐effective strategy for quantification is LFQ, as it requires neither metabolic nor chemical labeling. Samples are prepared and measured separately, and quantification is performed during data analysis. Peptides are detected in MS1 spectra based on their *m/z* ratio, retention time, and isotopic pattern. Identical peptides across runs are matched using precise retention time alignment and “match‐between‐runs” algorithms. Intensity‐based normalization and variance minimization compensate for technical variability.^[^ ^68^ ^]^ To achieve optimal accuracy and reproducibility, LFQ datasets should be acquired under identical LC‐MS/MS conditions and processed together (Figure 6C).

In organellar profiling, all three strategies can be used to generate high‐resolution organelle maps.^[^ ^76^ ^,^ ^77^ ^,^ ^80^, ^81^, ^82^ ^]^ For example, the original *dynamic organellar maps* (*DOMs*) method, where translocation events are aimed to be characterized between different states rather than a static organellar proteome map, was initially developed with SILAC but was later successfully adapted to LFQ, particularly in combination with DIA.^[^ ^76^ ^,^ ^81^ ^]^ Localization of Organelle Proteins by Isotope Tagging (LOPIT) and the improved successor hyperLOPIT are instead based on isobaric labeling.^[^ ^80^ ^,^ ^82^ ^]^ The optimal choice of strategy depends on several factors, including the specific research question (which organelle proteomes are of interest), sample availability and scalability (e.g., whether human primary samples are used), and the desired flexibility to integrate additional samples into existing datasets at a later stage.

### 4.3 Subsequent Bioinformatic Analysis

With the growing establishment of MS‐based organelle proteome profiling methods, the range of bioinformatic resources for data analysis has expanded considerably.^[^ ^77^ ^,^ ^152^ ^,^ ^153^ ^]^ These tools enable, among other functions, the annotation of proteins to specific organelles, the identification of similar distribution profiles, the characterization of changes in the localization patterns of individual proteins, and the analysis of coordinated changes in sets of proteins that collectively perform specific biological functions. Whereas in the early days of the technology each group had to develop its own analysis pipelines, more and more specialized R packages and interactive web interfaces are now available.^[^ ^30^ ^,^ ^76^ ^,^ ^80^, ^81^, ^82^ ^,^ ^154^, ^155^, ^156^ ^]^ These not only allow the analysis and visualization of spatial proteomics datasets but also facilitate their integration with functional data or other omics layers.

Despite these advances, the user accessibility of current spatial proteomics analysis tools still lags behind that of today's highly standardized global transcriptomic and proteomic pipelines because their effective use requires a more sophisticated understanding of the data structure and quality control metrics. The key criterion to make spatial proteomics bioinformatics tool accessible for nonexperts is that robust analysis should be feasible without requiring expert intervention and is readily feasible independent of a specific MS‐acquisition and quantification strategy. While default parameter settings may not always produce the absolute optimum in terms, they should ensure reliable, reproducible, and ideally readily interpretable results.

Spatial proteomics data present an inherent challenge from the outset: they are high‐dimensional datasets. Unlike conventional omics analyses, which often focus primarily on identifying proteins that are significantly up‐ or downregulated in condition A (e.g., untreated tumor cell line) compared to condition B (e.g., treated tumor cell line), spatial proteomics requires a more complex approach. Here, total protein abundance, the distribution across different organelles, and potential translocation events all need to be considered simultaneously.

#### 4.3.1 Global Data Visualization

Following preprocessing and data preparation, an important first step in the bioinformatic analysis is to visualize the overall structure of the dataset.^[^ ^157^ ^]^ Commonly used approaches for spatial proteomics data include dimensionality reduction techniques such as principal component analysis (PCA), based on linear transformation, and uniform manifold approximation and projection (UMAP), based on nonlinear transformation. Both methods reduce high‐dimensional datasets to two or three dimensions while retaining as much of the overall information as possible.^[^ ^158^ ^,^ ^159^ ^]^

**Figure 7:**
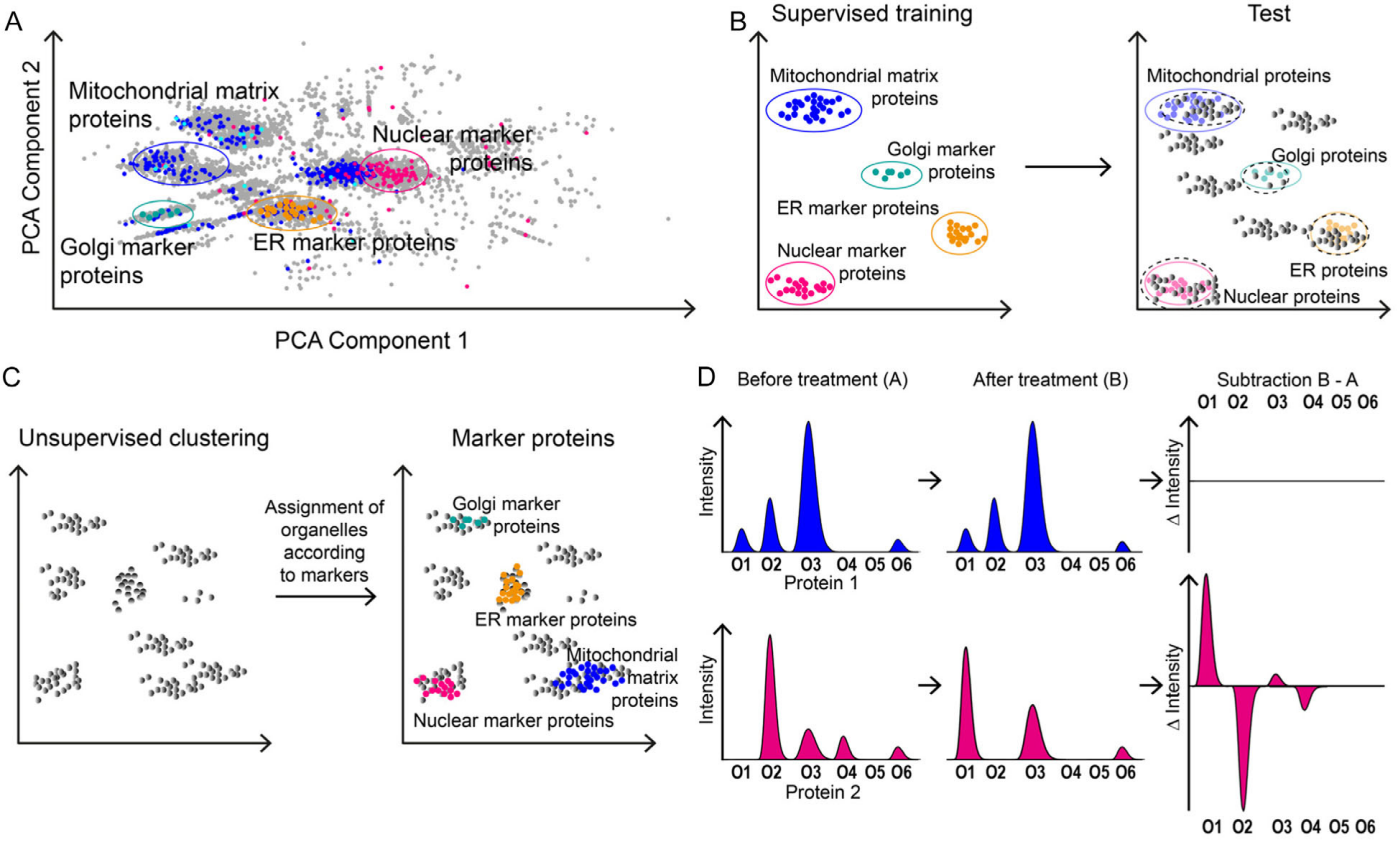
Bioinformatics analysis approaches for organellar profiling data. A) PCA analysis: Each protein is represented by a dot; known marker proteins for specific organelles are highlighted to reveal organelle‐associated clusters. B) Supervised clustering: A model is trained on representative organelle markers and then applied to the remaining proteins to predict their compartment assignments. C) Unsupervised clustering: Clusters are formed solely based on the data, and organelle identities are assigned afterward by locating known marker proteins within each cluster. D) Profile subtraction: Distribution profiles of proteins (e.g., Protein 1 and Protein 2) across organelles O1–O6 are compared before and after treatment. A shift in Protein 2's profile becomes apparent in the subtraction plot, whereas unchanged proteins yield a flat (null) subtraction profile.

In PCA, the first principal component (PC1) represents the direction in the data along which the greatest variance is observed and is typically plotted on the x‐axis. The second principal component (PC2), orthogonal to PC1, captures the second‐largest independent source of variance and is usually plotted on the *y*‐axis. In the resulting scatter plot, each point represents a protein, with its position determined by the values for PC1 and PC2. ^[^ ^146^ ^]^ Proteins with similar distribution profiles in the dataset appear close together. In successful organellar profiling experiments, proteins with a clear peak assignment to a specific organelle are expected to cluster together, enabling visual separation of the organelles (**Figure** **7**A).^[^ ^81^ ^]^

UMAP takes a different approach: it first calculates the similarity between all data points in high‐dimensional space to construct a neighborhood graph. This graph is then projected into a low‐dimensional space in a way that preserves local structure, that is, the relative distances to the nearest neighbors, as much as possible.^[^ ^159^ ^]^ The axes in a UMAP plot therefore have no direct ordering by explained variance and are not interpreted in the same way as PCA components. Instead, they simply represent the layout of the points in the 2D projection, with clusters of similar proteins appearing close to one another.

A variety of freely available software tools support both PCA and UMAP visualizations of organellar profiling data. While these methods are highly useful for identifying global patterns and cluster structures, they are inherently simplifications and therefore entail a degree of information loss. PCA or UMAP plots are thus well suited for detecting overall structures and relationships but are not designed to unambiguously assign individual proteins to specific compartments.^[^ ^77^ ^]^

#### 4.3.2 Compartment Assignment

When the goal is to determine in which compartment individual proteins are localized, the outcome of the bioinformatic analysis is typically a list that assigns each protein to a specific organelle. This is most reliable for proteins that, in the given biological context, are unambiguously associated with a single compartment. Prediction of compartment assignments can be performed using either supervised or unsupervised clustering approaches.^[^ ^77^ ^]^ Overall, high‐quality input data remain the critical prerequisite for reliable classification and have a stronger impact on prediction performance than the specific classification method that has been applied.^[^ ^155^ ^]^

In supervised clustering, predefined marker proteins that are clearly assigned to an organelle are used as a training dataset for a machine learning model. Based on the distribution profiles of these markers, the model is generated and then applied to all other proteins (Figure 7B).^[^ ^81^ ^]^ Supervised clustering approaches include, for example, support vector machines (SVMs), k‐nearest neighbors (kNN), and random forests (RFs) that differ conceptually how class boundaries are defined.^[^ ^77^ ^]^ SVMs determine an optimal separating surface (hyperplane) that divides proteins from different organelles in multidimensional space. kNN, in contrast, assigns each protein according to the majority class among its closest neighbors, whereas RFs aggregate the outcomes of multiple decision trees to improve prediction robustness. Each approach has distinct advantages: SVMs for accuracy, kNN for interpretability, and RFs for handling nonlinear feature interactions. The quality of the model depends critically on the reliability and context‐appropriateness of the markers. For example, a curated list of 581 marker proteins with very high predictive value for human cells has been compiled across multiple studies.^[^ ^30^ ^]^ However, these markers are not necessarily transferable to other biological systems or specific physiological contexts. Enzymes of the TCA cycle, for instance, are frequently used as mitochondrial markers. If these proteins are predominantly located in the nucleus in a specific context, such as during the maternal‐to‐zygotic transition, their use as mitochondrial markers would lead to incorrect predictions.

In unsupervised clustering, the measured distribution data alone are used to group proteins into clusters, without prior marker input (Figure 7C).^[^ ^160^ ^]^ The organelle represented by each cluster is assigned only after clustering, based on the identity of its members. This approach has the potential to reveal previously unknown compartment associations or novel substructures. Unsupervised clustering methods include approaches such as k‐means clustering and model‐based techniques like the Gaussian mixture model (GMM).^[^ ^77^ ^]^ In k‐means clustering, proteins are grouped into *k* clusters based on the similarity of their quantitative distribution profiles. This approach is widely used for exploratory analysis but assumes that clusters are approximately spherical and of similar variance. In contrast, GMM assumes that the data are generated from a mixture of underlying Gaussian distributions, each representing a potential cluster. Initially, neither the cluster membership of each protein nor the parameters of these distributions (e.g., means and variances) are known. To estimate these parameters and assign proteins probabilistically, the Expectation–Maximization algorithm is typically applied. This iterative procedure alternates between two steps. In the *Expectation* step, the probability that each data point belongs to each cluster is calculated based on the current parameter estimates. In the subsequent *Maximization* step, the distribution parameters are updated to maximize the likelihood of the observed data given these probabilistic assignments. These steps are repeated until convergence, yielding stable cluster memberships and optimized model parameters.

Ultimately, both approaches rely on marker sets—supervised clustering uses them directly for model training, while unsupervised clustering uses them post hoc for cluster annotation. Markers are also essential for assessing prediction accuracy. It is important to note that prediction models always produce probability distributions. These are well suited for identifying general trends, but for precise conclusions regarding individual proteins, it is advisable to examine their distribution profiles in detail.

Both approaches have limitations when it comes to predicting the localization of proteins that are associated with more than one compartment—a situation estimated to apply to at least 30% of proteins, and often to an even higher proportion.^[^ ^77^ ^,^ ^161^ ^]^ For such proteins, an unambiguous assignment to a single compartment is inherently not possible. However, their distribution profiles can still change under different conditions.^[^ ^77^ ^]^ For example, a protein may be approximately evenly distributed between two compartments under steady‐state conditions but, following a perturbation, such as drug treatment of tumor cells or during a developmental transition in stem cells, may display a marked preference for one compartment.

These shifts in distribution profiles are of particular interest because they can provide early insights into the molecular mechanisms that contribute to the emergence of a specific phenotype at the protein level. A common strategy for detecting such changes is profile subtraction, in which the distribution profile under condition A is subtracted from that under condition B (Figure 7D). Proteins without substantial changes show no pronounced peaks in the resulting difference profile, whereas proteins with strong relocalization events display prominent deviations.^[^ ^76^ ^,^ ^81^ ^]^ In practice, it is advisable to first systematically identify proteins that exhibit statistically significant changes in their distribution profiles and then, in a second step, examine in detail which compartment the redistribution favors.

#### 4.3.3 Examples for Computational Frameworks for Spatial Proteomics Data Analysis

Among computational frameworks for spatial proteomics data analysis, the R/Bioconductor ecosystem has become particularly powerful in terms of data analysis but also visualization.

pRoloc was introduced in 2014 as a comprehensive framework for the analysis and visualization of quantitative, MS‐based spatial proteomics data.^[^ ^155^ ^]^ It provides structured methods for protein localization prediction, cluster identification, and data exploration through a variety of supervised and unsupervised machine learning algorithms. For initial data processing, pRoloc relies on the companion package MSnbase, which organizes quantitative information from fractionation experiments into an MSnSet object that forms the core data structure for downstream analysis.^[^ ^162^ ^,^ ^163^ ^]^ Although pRoloc can integrate data from external preprocessing tools such as MaxQuant^[^ ^150^ ^]^ and DIA‐NN,^[^ ^70^ ^]^ these tabular outputs must first be reformatted into the expected MSnSet structure containing protein intensities, metadata, and experimental annotations. pRoloc supports a wide range of algorithms, including unsupervised approaches such as GMM and supervised classifiers such as SVMs, enabling both exploratory analysis and probabilistic protein localization. A key methodological advance came with the introduction of Bayesian uncertainty modeling in later work, which allows localization probabilities to be treated as posterior estimates derived from the combination of prior biological knowledge and observed quantitative data.^[^ ^164^ ^]^ This approach provides a formal measure of confidence for each protein's predicted localization. While pRoloc does not explicitly label proteins with multiple subcellular localizations, a broad posterior distribution or high assignment uncertainty can indicate potential dual or dynamic localization. Overall, pRoloc offers an open and modular architecture within the R environment that supports flexible workflow customization and integration with other bioinformatic packages. It includes powerful visualization tools for intuitive data exploration but requires basic proficiency in R and manual data conversion when external preprocessing pipelines are used. Moreover, dynamic or condition‐dependent relocalization is not modeled explicitly but can be inferred indirectly through increased uncertainty in the probabilistic output.

Another computational framework specifically developed to detect protein translocation events is TRANSPIRE, introduced in 2020.^[^ ^165^ ^]^ This platform integrates quantitative proteomics data with spatial localization information to predict changes in subcellular protein distribution. It generates synthetic translocation profiles by combining distribution patterns of organelle marker proteins from two experimental conditions (e.g., nucleus in condition A and cytoplasm in condition B). Analogous combinations of markers from the same organelle serve as negative controls, representing stable localizations. These synthetic examples are used to train a Stochastic Variational Gaussian Process classifier, a nonparametric, Bayesian model capable of learning nonlinear relationships between quantitative distribution profiles and localization classes without assuming predefined distribution shapes. Once trained, the model predicts the probability of translocation for each protein across all possible compartment pairs and can annotate results with cotranslocating proteins, protein complexes, and Gene Ontology terms, thereby providing functional context. While TRANSPIRE offers strong potential for identifying and functionally characterizing protein relocalization events, it requires external data preprocessing and format conversion, relies on externally provided curated organellar marker lists, and its use demands intermediate computational expertise.

A recently developed framework extending the concept of TRANSPIRE is TransGCN, a method for inferring protein translocation based on a semi‐supervised graph convolutional network (GCN) architecture, which was introduced 2024.^[^ ^166^ ^]^ GCNs integrate both the intrinsic features of proteins and the relational structure among them, representing proteins as nodes connected by edges that encode similarity or functional association. The semi‐supervised design allows the network to learn from a subset of labeled proteins with known subcellular localizations while propagating this information through the graph to infer labels for unclassified proteins. By aggregating information from neighboring nodes, the model refines each protein's representation according to its network context, which is particularly valuable in spatial proteomics datasets that are heterogeneous and only partially annotated. To train the model, TransGCN generates a synthetic dataset of established organellar markers and known translocation events by differential matching, combining, for example, the profile of a protein localized to compartment X in condition 1 with that of a protein localized to Y in condition 2 to simulate an X→Y translocation, following a strategy similar to TRANSPIRE. Proteins with reliable localizations are selected using a Z‐score test to estimate the probability of association with each fraction. In the second step, TransGCN constructs a hybrid graph linking the synthetic and real datasets using mutual nearest neighbor matching, a symmetric variant of kNN that enhances robustness for data integration. Within this graph, distance‐based features and probabilistic metrics are computed to capture relationships between proteins. Finally, the semi‐supervised GCN leverages both labeled (synthetic) and unlabeled (experimental) data to predict protein localizations and translocation events. This framework provides a powerful and flexible approach for identifying dynamic localization changes from high‐dimensional spatial proteomics data.

In addition to R‐based frameworks for spatial proteomics analysis, several alternative applications have been developed to support users without prior expertise in proteomics data analysis or programing. These tools emphasize accessibility through graphical user interfaces that guide users interactively through data processing and visualization steps.

One such web‐based platform is DOM‐ABC, introduced in 2023 for the analysis and quality assessment of DOMs.^[^ ^76^ ^]^ DOM‐ABC enables in‐depth benchmarking, quality control, and visualization of organellar profiling data within an intuitive browser‐based environment. Users can upload proteomics datasets preprocessed with tools such as MaxQuant,^[^ ^150^ ^]^ configure analysis parameters via dropdown menus, and generate interactive plots for exploratory visualization and interpretation. By combining a structured, step‐by‐step workflow with an accessible GUI, DOM‐ABC offers a low‐barrier entry point for researchers new to spatial proteomics analysis. However, its flexibility is limited compared to script‐based frameworks: manual adjustment of data processing steps is restricted, and seamless integration into customized analysis pipelines is limited.

For broader data exploration and integration, the Perseus platform provides a versatile graphical environment for multivariate and statistical analysis of large‐scale proteomics datasets.^[^ ^167^ ^]^ The software integrates seamlessly with MaxQuant,^[^ ^150^ ^]^ outputs but is also compatible with data processed by a variety of other tools such as DIA‐NN,^[^ ^70^ ^]^ and includes modules for normalization, missing‐value imputation, clustering, and differential abundance testing. All analytical steps are represented within an intuitive workflow diagram, allowing users to visualize the sequence of operations and maintain transparency throughout data processing. Each transformation generates a new matrix while preserving the original dataset, minimizing the risk of data loss and making Perseus particularly suitable for users new to quantitative proteomics. Although not specifically optimized for spatial proteomics, Perseus is well suited for downstream processing of abundance profiles derived from any kind of proteomics experiments. With additional programing expertise, its modular plugin architecture allows extension and integration with R‐based pipelines, thereby bridging graphical and script‐based analytical environments.

Altogether, the growing ecosystem of computational frameworks illustrates the rapid evolution of bioinformatic tools for spatial proteomics. These resources collectively enable more reproducible, quantitative, and interpretable analyses of protein localization dynamics across diverse experimental designs. However, achieving greater interoperability, automation, and standardization remains a key challenge. Future developments will likely focus on integrating these complementary approaches into unified, user‐accessible workflows that combine analytical flexibility with biological interpretability.

## 5 Spatial Proteomics for Clinical Applications and Future Directions

The complex organizational structure of a tissue is fundamental to its function and plays a decisive role in disease initiation and progression.^[^ ^168^ ^]^ For instance, immune cell infiltration can profoundly remodel the tumor microenvironment to promote its plasticity and heterogeneity.^[^ ^169^ ^]^ In this context, spatial proteomics has emerged as a powerful tool for clinical research, enabling the elucidation of pathomechanisms, the identification of predictive and prognostic biomarkers, and the discovery of therapeutic targets across diverse indications, including cancer, inflammatory and infectious diseases, and cardiovascular disorders.^[^ ^170^, ^171^, ^172^, ^173^, ^174^ ^]^ Since many of the available platforms are readily compatible with both formalin‐fixed, paraffin‐embedded (FFPE) and fresh‐frozen tissues, they are well suited for large cohort studies.

In current clinical practice, in situ protein detection still relies mainly on antibody‐based, targeted approaches directed against predefined epitopes. Fluorescence‐based multiplexed imaging is widely employed owing to the broad availability of antibodies, imaging platforms, and standardized workflows. However, spectral overlap and nonspecific antibody binding constrain the number of proteins that can be visualized simultaneously.^[^ ^175^, ^176^, ^177^ ^]^ To overcome some of these limitations, cyclic immunofluorescence (CyCIF) strategies such as codetection by indexing or CyCIF use fluorophore‐ or DNA‐barcoded antibody conjugates in iterative staining and imaging cycles while preserving spatial context.^[^ ^176^ ^,^ ^178^ ^]^ In addition, high‐plex tissue imaging approaches such as IMC and MIBI extend multiplexing capacity even further, enabling simultaneous detection of more than 30 protein markers on a single section. These methods are now widely applied for risk stratification, therapy‐response monitoring, and mechanism‐based biomarker discovery in clinical material.^[^ ^126^ ^,^ ^179^ ^,^ ^180^ ^]^

An emerging class of spatial proteomics techniques uses DNA barcoding and sequencing for molecular readout rather than imaging, which enables signal amplification and is therefore particularly interesting for clinical applications with low input samples. In these approaches, protein abundance is translated into sequencing‐readable DNA barcodes via antibody‐derived DNA tags (ADTs), enabling quantitative and highly multiplexed mapping of protein localization within tissue architecture. These techniques can be also combined with complementary readouts like immunofluorescence imaging and bridge different omics layers by simultaneous comapping of the transcriptome.^[^ ^181^, ^182^, ^183^, ^184^, ^185^ ^]^ The concept originated from antibody sequencing and cellular indexing of transcriptomes and epitopes by sequencing (CITE‐seq), which first introduced ADTs to quantify protein abundance in single cells via NGS.^[^ ^186^ ^,^ ^187^ ^]^ Building on this principle, Deterministic Barcoding in Tissue sequencing adapted DNA barcoding to the tissue plane through a microfluidic A×B grid, achieving ≈10 µm spatial resolution for simultaneous transcriptome and protein profiling of 20–100 proteins, revealing complex microarchitectures such as endothelial and epithelial networks and developmental patterning domains.^[^ ^181^ ^]^ Subsequent innovations have expanded both scale and sensitivity. Spatial‐CITE‐seq and Stereo‐CITE‐seq increased multiplexing to ≈200–300 proteins while with a pixel size of 25 µm and below, enabling high‐resolution molecular reconstruction of immune zonation and tumor microenvironments.^[^ ^182^ ^,^ ^183^ ^]^ Array‐based implementations such as spatial prOtein and transcriptome sequencing and spatial multiomics integrated ADT‐capture into barcoded slide arrays, facilitating automation and high throughput albeit with moderate spatial resolution (≈50 µm).^[^ ^184^ ^,^ ^185^ ^]^ More recently, Molecular Pixelation (MPX) eliminated predefined grids by using combinatorial “molecular pixels,” allowing subcellular (≈1‐2 µm) inference of protein localization and proximity networks.^[^ ^188^ ^]^ Finally, DBiT‐plus integrates sequencing‐based barcoding with imaging proteomics to combine quantitative molecular depth with high spatial precision.^[^ ^189^ ^]^

Although recently developed antibody‐based platforms enable high levels of multiplexing, they cannot provide a truly comprehensive view of the proteome, as they rely on the preselection of target proteins. Even methods capable of detecting over 100 protein markers capture only a small subset of the >10 000 proteins expressed in a typical mammalian cell. In contrast, ultrasensitive LC‐MS/MS, particularly when combined with imaging‐guided workflows such as DVP, offers the potential for deep, untargeted proteome quantification at high spatial resolution in clinical tissue samples.^[^ ^133^ ^,^ ^134^ ^]^ Another important recent advance toward volumetric spatial proteomics is 3D imaging of solvent‐cleared organs and biopsy samples profiled by MS (DISCO‐MS).^[^ ^177^ ^]^ DISCO‐MS combines tissue clearing, high‐resolution microscopy, and targeted LCM with ultrasensitive LC‐MS/MS to achieve 3D proteomic profiling of intact biological specimens. Clinically, the method holds considerable promise. By allowing proteome‐wide analysis of defined anatomical regions from archived or fresh tissue, it can link molecular alterations directly to complex structural lesions or microenvironmental features.

Recent advances in omics technologies now enable single‐cell‐resolved integration of multiple molecular layers, encompassing genomic, epigenomic, transcriptomic, and proteomic features.^[^ ^190^ ^]^ This multiomics integration holds great promise for precision medicine, as it can uncover regulatory networks that drive disease mechanisms, identify novel therapeutic targets, and ultimately inform clinical decision‐making.^[^ ^191^ ^]^ Looking ahead, future efforts will also focus on comprehensive profiling of subcellular and organelle‐specific proteomes in clinical samples, as such holistic spatial analyses provide mechanistic insight into the molecular alterations underlying disease progression.^[^ ^78^ ^,^ ^122^ ^,^ ^192^ ^]^

A major challenge for organelle profiling with clinical material is the limited sample amount, which directly impacts organelle separation. While centrifugation‐based fractionation protocols have proven robust for cultured cells,^[^ ^76^ ^,^ ^77^ ^,^ ^82^ ^,^ ^140^ ^,^ ^161^ ^]^ they require considerable technical expertise and are prone to losses and contamination when starting with small numbers of cells. Microscopy‐guided segmentation approaches are emerging now as an alternative, as they offer the possibility to label and thereby enrich proteins from a specific compartment even from small amounts of starting material.^[^ ^116^ ^]^ However, these methods inherently allow the analysis of only limited compartments or structures per experiment. This makes them less suited for monitoring translocations between organelles in parallel.

From our perspective, antibody‐based organelle enrichment for multiorganelle proteome profiling with clinical specimen is particularly promising for several reasons. First, the affinity enrichment with stringent washing (e.g., via antibody‐coated magnetic beads) improves the signal‐to‐noise ratio and reduces contamination, which is critical when working with low‐input material despite major advances in data analysis.^[^ ^155^ ^]^ Second, the rapid workflows does not require long incubation times or specialized microscopy equipment. And last, it holds high potential for automation, a prerequisite for routine high‐throughput applications. The current bottleneck is the availability of high‐quality affinity reagents targeting endogenous organelle surface markers. Many published protocols rely on genetically introduced epitopes (e.g., GFP, HA),^[^ ^30^ ^,^ ^93^ ^,^ ^94^ ^]^ which are not feasible for clinical samples. Recent advances in AI‐assisted antibody design and the development of aptamers (via SELEX) or aptamer‐like DNA origami scaffolds open new opportunities,^[^ ^193^ ^]^ though their clinical readiness varies and requires rigorous validation. Nevertheless, targeted enrichment of specific organelles can be accomplished with only a small set of highly selective antibodies or other applicable reagents, in contrast to multiplexed antibody‐based mapping approaches that require large reagent panels to profile hundreds of proteins simultaneously.

From an MS analysis perspective, clinical material is not inherently limiting: modern instruments enable low‐input and even single‐cell analyses.^[^ ^133^ ^,^ ^194^ ^,^ ^195^ ^]^ However, classical organelle‐resolved profiling (involving multiple fractions) still generally requires more material per fraction than whole‐proteome measurements.^[^ ^79^ ^]^ Low‐input workflows, featuring careful pooling, loss‐minimizing handling, and optimized LC‐MS/MS methods, can reduce these requirements and still deliver high‐resolution organellar maps, provided that separation is clean and reproducible.

In our view, there remains also a need for improved bioinformatic analysis pipelines tailored to clinical datasets, where mechanistic insights are the primary goal. Global distribution shifts are informative but must be resolved to protein‐specific relocalization events. Heuristics such as difference/subtraction profiles (condition B minus A) can help prioritize significant shifts. Since marker behavior may deviate from expectations in pathological contexts, thereby reducing the predictive power of trained models, marker‐robust strategies are crucial. This particularly includes outlier detection, model‐free cluster annotation, integration with functional proteome datasets like phospho‐proteomics, and context‐specific marker sets. Independent orthogonal validation remains essential, with immunofluorescence and complementary subcellular fractionation/immunoblotting being established approaches.

Overall, progress in the field of organellar profiling approaches for clinical applications will depend on three key factors: 1) low‐input, automatable sample preparation with reliable affinity reagents for endogenous targets, 2) standardized, marker‐robust bioinformatic pipelines with clear QC metrics that are also accessible and interpretable for non specialists, and 3) rigorous orthogonal validation of prioritized relocalizations. Under these conditions, we expect that organellar‐profiling spatial proteomics workflows will have a direct impact on diagnostics and therapeutic target discovery and contribute to precision medicine.

## Conflict of Interest

The authors declare no conflict of interest.

## Author Contributions

**Franziska Traube**: conceptualization (lead); funding acquisition (lead); writing original draft (equal); writing—review and editing (supporting). **Chiara Bernardini**: writing—original draft (equal); writing—review and editing (equal). **Maike Däther**: writing—original draft (equal); writing—review and editing (equal). **Chiara Bernardini** and **Maike Däther** contributed equally on this work.
